# Wide-Field Choroidal Thickness Analysis after Half-Fluence Photodynamic Therapy Combined with Intravitreal Aflibercept Injection in Pachychoroid Neovasculopathy

**DOI:** 10.3390/jcm13061608

**Published:** 2024-03-11

**Authors:** Yosuke Fukuda, Shoji Notomi, Satomi Shiose, Yusuke Maehara, Kohei Kiyohara, Sawako Hashimoto, Kumiko Kano, Keijiro Ishikawa, Toshio Hisatomi, Koh-Hei Sonoda

**Affiliations:** 1 Department of Ophthalmology, Graduate School of Medical Sciences, Kyushu University, 3-1-1 Maidashi, Higashi-ku, Fukuoka 812-8582, Japan; fukuda.yosuke.144@m.kyushu-u.ac.jp (Y.F.); shiose.satomi.705@m.kyushu-u.ac.jp (S.S.); kiyohara.kohei.685@s.kyushu-u.ac.jp (K.K.); hashimoto.sawako.195@m.kyushu-u.ac.jp (S.H.); kano.kumiko.599@m.kyushu-u.ac.jp (K.K.); ishikawa.keijiro.609@m.kyushu-u.ac.jp (K.I.); k.sonoda.a74@m.kyushu-u.ac.jp (K.-H.S.); 2 Department of Ophthalmology, Fukuoka University Chikushi Hospital, 1-1-1 Zokumyouin, Chikushino, Fukuoka 818-8502, Japan; t.hisatomi.qk@fukuoka-u.ac.jp

**Keywords:** age-related macular degeneration, pachychoroid neovasculopathy, photodynamic therapy, anti-vascular endothelial growth factor therapy, ultra-wide-field optical coherent tomography

## Abstract

**Background**: Pachychoroid neovasculopathy (PNV) is a pachychoroid-spectrum disease. As blood circulation throughout the choroid may be involved in PNV pathogenesis, analysis using ultra-wide-field (UWF) fundus imaging is crucial. We evaluated choroidal thickness after half-fluence photodynamic therapy (PDT) combined with intravitreal aflibercept injection for PNV using UWF swept-source optical coherence tomography. **Methods**: Seventeen eyes with PNV that underwent half-fluence PDT with an adjuvant single intravitreal aflibercept injection were analyzed. To compare choroidal thicknesses in the central and peripheral choroids, we set subfields <3, <9, and 9–18 mm from the fovea. The <9 and 9–18 mm subfields were divided into four quadrants. **Results**: Choroidal thickness in each subfield decreased significantly after half-fluence PDT (*p* < 0.001); this reduction was more pronounced in the central area. We also investigated the relationship between the dominant side of the deep choroidal veins that harbor choroidal vein efflux from the macula. When choroidal thickness in the supratemporal and infratemporal 9 mm subfields were evaluated, the ratio of choroidal thickness reduction was not significantly different between the dominant and non-dominant sides. The dominant side was not associated with the extent of choroidal thickness reduction in PNV. **Conclusions**: Half-fluence PDT caused thinning of the entire choroid, especially in the central area, in PNV.

## 1. Introduction

Pachychoroid neovasculopathy (PNV), a type of pachychoroid-spectrum disease (PSD), is characterized by macular neovascularization (MNV) being associated with choroidal thickening, dilated choroidal vessels, or choroidal vascular hyperpermeability (CVH) [1,2,3,4]. Similar to central serous chorioretinopathy (CSC), a thickened choroid observed in PNV is more pronounced in the posterior pole than in the periphery [5,6,7]. While CSC is associated with pachychoroid-driven leakage through the breakdown of the retinal pigment epithelium (RPE) barrier, similar choroidal pathology may also lead to the development of MNV [8]. PNV is associated with MNV; however, its clinical manifestations and genetic features are largely different from those of neovascular age-related macular degeneration (AMD) [9,10,11].

Although anti-VEGF therapy is effective against PNV, several studies have shown that patients require frequent clinical visits and repeated intravitreal injections [12,13,14]. Photodynamic therapy (PDT) has been demonstrated to resolve the subretinal fluid (SRF) in CSC [15,16], suggesting that PDT could be effective against PNV. Indeed, the combination of half-dose/fluence PDT with anti-VEGF therapy has been shown to reduce the need for frequent injection regimens for PNV [17,18]. 

Dysregulation of choroidal circulation, which normally drains through the vortex vein ampulla, has been suggested to play a role in the pathology of PSD [19]. Therefore, assessing choroidal thickness, including that of the peripheral choroid, is important in PSD studies. A study showed that eyes affected by CSC demonstrated a higher frequency of vertical asymmetry in the running pattern of the deep choroidal veins compared to that in those of healthy participants [20]. Although this asymmetry is observed in healthy individuals, it is believed to play a role in the pathology of PSD. 

Ultrawide-field optical coherence tomography (UWF-OCT) facilitates the measurement of choroidal thickness in the peripheral regions. Our previous study demonstrated that the choroidal thickness in PNV exceeds that of typical age-related macular degeneration (AMD) even after adjusting for axial length and age [5]. Although recent studies have documented changes in choroidal thickness following PDT in CSC using UWF-OCT [21,22,23], such evidence remains limited for PNV. This study aimed to analyze variations in choroidal thickness after half-fluence PDT for PNV using UWF-OCT.

## 2. Materials and Methods

### 2.1. Patients

This retrospective study was approved by the Institutional Review Board of Kyushu University, Fukuoka, Japan, and adhered to the tenets of the Declaration of Helsinki. All data were anonymized prior to analysis. We included patients with treatment-naïve PNV who visited Kyushu University Hospital between March 2021 and June 2023 and underwent half-fluence PDT with 6 mg/m^2^ verteporfin. 

The diagnosis of PNV was based on the absence of large (>125 μm) drusen in the macular area within a 6 mm diameter circle centered on the fovea. On the other hand, the existence of pachydrusen outside the macular area was not excluded. In addition, PNV diagnosis was defined by the presence of the following factors: (1) type 1 MNV, as visualized using OCT angiography; (2) pathologically dilated outer choroidal vessels on OCT and ICGA images; and (3) regional CVH on ICGA images (performed in all enrolled patients). The identification of pachyvessels was determined by three retinal experts (S.S., K.K., and S.N.). Patients with polypoidal lesions and those with a history of treatment for PNV were excluded. All patients received an adjuvant single intravitreal injection of 2.0 mg/0.05 mL aflibercept (EYLEA^®^; Regeneron Pharmaceutical Inc. and Bayer, Tarrytown, NY, USA) prior to PDT. Half-fluence PDT was performed 1 week after administering the intravitreal injection of aflibercept. Fifteen minutes after initiating intravenous verteporfin infusion, a 689 nm laser was applied for 83 s to deliver 25 J/cm^2^. The greatest linear dimension (GLD) was determined to cover the abnormal vascular network.

At the initial presentation, all patients underwent a comprehensive ophthalmic examination of both eyes, including best-corrected visual acuity (BCVA) using the Landolt chart, slit-lamp biomicroscopy, intraocular pressure, color fundus photography covering the 45-degree posterior retina, fluorescein/indocyanine-green angiography, near-infrared reflectance, fundus autofluorescence (HRA-II; Heidelberg Engineering, Dossenheim, Germany), spectral-domain OCT (Spectralis HRA + OCT or CirrusTM 5000 HD-OCT; Zeiss, Dublin, CA, USA), and UWF swept-source OCT (Xephilio OCT-S1; Canon Medical Systems, Tokyo, Japan). OCT angiography was performed using the AngioVue Imaging System (Optovue Inc., Fremont, CA, USA). All patients underwent a follow-up examination using UWF swept-source OCT 3 months after half-fluence PDT. If a patient developed PNV in both eyes, the eye that occurred first was considered for evaluation.

### 2.2. Evaluation of Choroidal Thickness via UWF Swept-Source OCT

Central and peripheral choroidal thicknesses were analyzed as previously described [5,6]. Briefly, we acquired three-dimensional volume data with vertical dimensions of 20 mm, horizontal dimensions of 23 mm, and scan depths of 5.3 mm using UWF swept-source OCT. For segmentation of the choroid, we set the choroidal thickness as the vertical distance from the Bruch’s membrane to the chorioscleral interface. Segmentation was automatically performed using the built-in software (Xephilio OCT-S1, ver. 2.0) and manual correction when required. For a more accurate evaluation of the wide-field choroidal thicknesses, we performed an automatic real-shape correction on the acquired OCT images using the software provided by Canon Inc. (Tokyo, Japan) (US patent 9149181 B2) [24]. Automatic measurements of choroidal thicknesses were performed using the OCT Research Tool (Canon Inc.).

**Figure 1:**
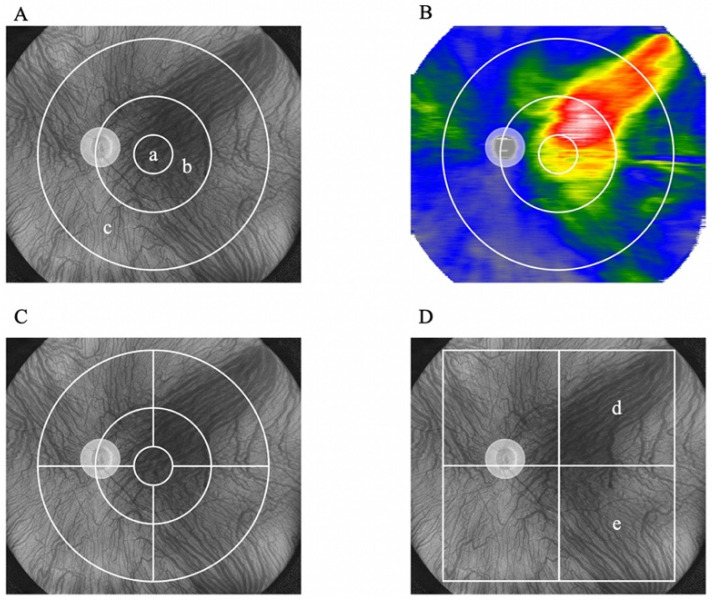
Choroidal thickness map and subfields. Representative images of the subfields and choroidal thickness maps obtained using UWF-OCT. (**A**) Three subfields, 3 mm (a), 9 mm (b), and 9–18 mm (c), are illustrated on an en-face UWF-OCT image. (**B**) Representative image of the choroidal thickness map calculated from the Bruch’s membrane to the chorioscleral interface in UWF OCT b-scans. (**C**) The 3–9 and 9–18 mm subfields were divided into four subfields. (**D**) Four square subfields were set, and the supratemporal (d) and infratemporal (e) subfields were analyzed. UWF-OCT: ultrawide-field optical coherence tomography.

To compare the central and peripheral choroidal thicknesses, we set a grid consisting of three circles with diameters of 3, 9, and 18 mm, centered on the fovea. Three subfields were set up in this study as follows: (a) <3 mm, (b) <9 mm, and (c) 9–18 mm (Figure 1A,B). Furthermore, to examine changes in choroidal thickness before and after treatment in detail, we divided the 3–9 mm subfield and the 9–18 mm subfield into the following four quadrants: supratemporal, infratemporal, supranasal, and infranasal (Figure 1C).

**Figure 2:**
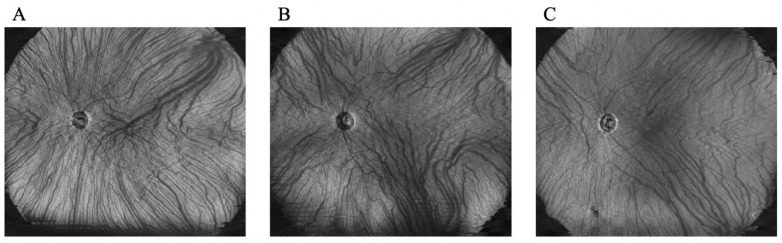
Symmetrical and asymmetrical patterns of deep choroidal veins and the vortex vein in en-face images. Representative cases from each of the following three groups: upper dominant (**A**), lower dominant (**B**), and asymmetrical (**C**).

Two authors (S.N. and Y.F.) evaluated the presence of asymmetry and the dominant and non-dominant sides of the vortex veins for each patient with PSD. The dominant or non-dominant sides of the vortex vein were determined by the deviation of the temporal horizontal watershed zone and were categorized into asymmetrical (upper or lower dominants) and symmetrical groups (Figure 2). To assess choroidal thickness, including areas near the vortex vein ampulla, we set a 9 mm^2^ area located supra/inferotemporally from the fovea, with the fovea as one of its corners (Figure 1D). Changes in choroidal thickness were compared between the dominant and non-dominant sides (Figure 1D). 

### 2.3. Statistical Analyses

Choroidal thickness in each subfield was measured before and 3 months after PDT. The patients were divided into two groups according to the presence of persistent SRF. We used the Mann–Whitney U test or Fisher’s exact test for analyzing differences in patient background or choroidal thickness between the groups. The Wilcoxon signed-rank test was performed to compare changes in choroidal thickness and BCVA after treatment. Statistical significance was set at a *p*-value of 0.05.

## 3. Results

### 3.1. Characteristics of the Enrolled Patients

Sixteen eyes from sixteen patients (including nine men and seven women) were analyzed in the study. The mean ± standard deviation (SD) age was 64.9 ± 12.9 years. The mean ± SD axial length was 24.4 ± 1.1 mm, and the mean ± SD GLD was 3506 ± 934 μm. The mean ± SD baseline BCVA was 0.15 ± 0.20 logarithm of the minimum angle of resolution (logMAR), and the mean ± SD central retinal thickness was 336.0 ± 76.0 μm. There were eight eyes (50.0%) in the upper dominant group, three in the lower dominant group, and five in the asymmetrical group (Table 1). SRF was present before treatment in all cases. No hemorrhage or subretinal hyperreflective material was observed, and there were no cases of peripapillary pachychoroid syndrome or peripheral exudative hemorrhagic chorioretinopathy.

### 3.2. Treatment Outcome and Factors Related to the Presence of SRF after Treatment

Of sixteen eyes, residual or recurrent SRF was observed in seven eyes 3 months after treatment. In one eye, SRF was observed within the first 3 months after treatment. However, in all cases, including this one, no additional PDT or an additional injection of an anti-VEGF drug was administered within the initial 3 months post-treatment. Additionally, in one eye, SRF was observed 1 month after treatment but disappeared without additional treatment 2 months after treatment.

**Figure 3:**
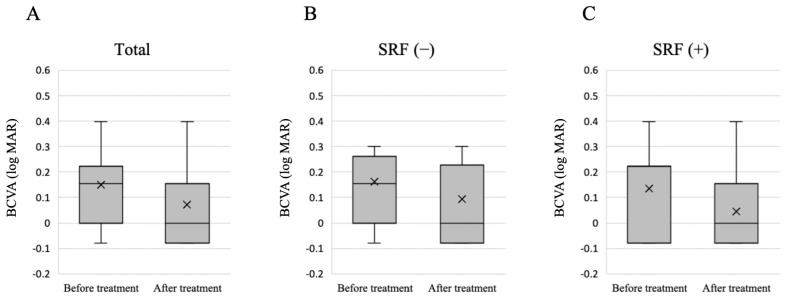
Changes in BCVA before and 3 months after treatment. (**A**) Changes in BCVA in all 16 eyes. The mean BCVA significantly improved after the treatment (*p* = 0.02, Wilcoxon signed rank test). (**B**) Changes in BCVA in nine eyes with no SRF at 3 months after treatment. No significant difference in BCVA was observed before and after treatment (*p* = 0.07). (**C**) Changes in BCVA in seven eyes with SRF at 3 months after treatment. No significant difference in BCVA was observed before and after treatment (*p* = 0.09). The cross symbol represents the mean marker. BCVA: best-corrected visual acuity; SRF: subretinal fluid; logMAR: logarithm of the minimum angle of resolution.

**Figure 4:**
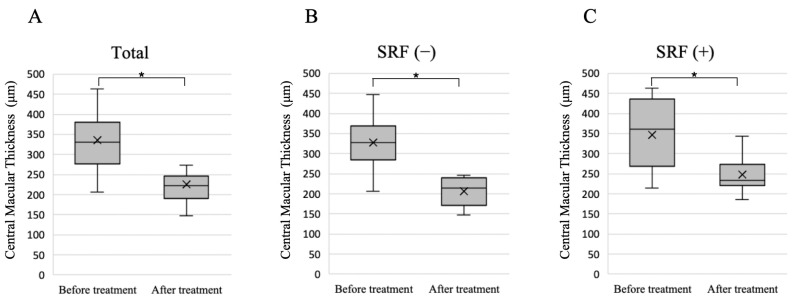
Changes in central macular thickness before treatment and 3 months after treatment. (**A**) Changes in central macular thickness in all 16 eyes. (**B**) Changes in central macular thickness in nine eyes with no SRF 3 months after treatment. (**C**) Changes in central macular thickness in seven eyes with SRF 3 months after treatment. Significant differences in central macular thickness were observed before and after treatment in all groups. The cross symbol represents the mean marker. * *p* < 0.001, Wilcoxon signed-rank test. SRF: subretinal fluid.

We examined factors related to the presence or absence of SRF 3 months after treatment; however, no significant differences in patient background were observed (Table 2). Despite the absence of significant differences, the group in which SRF was observed three months after treatment tended to be older before treatment (*p* = 0.09). Furthermore, there was no significant difference in BCVA before and after treatment, and this did not vary according to the presence or absence of SRF 3 months after treatment (Figure 3). No patients experienced an increase in BCVA of 0.2 or more. Overall, CMT decreased significantly after treatment, and this decrease was not dependent on the presence or absence of SRF 3 months after treatment (Figure 4).

### 3.3. Choroidal Thickness before and after Treatment

A significant decrease in choroidal thickness was observed after treatment in all areas (*p* < 0.001; Table 3). Additionally, we investigated whether the choroid was more likely to become thinner in the posterior (9 mm subfield) or peripheral (9–18 mm subfield) areas (Figure S1). Results showed that choroidal thickness significantly decreased in the 9 mm subfield compared to that in the 9–18 mm subfield (*p* = 0.002; Table 4). Although there was no statistically significant difference, the choroid in the 9–18 mm subfield before treatment was thinner in the group with SRF at 3 months after treatment (*p* = 0.09; Table 5).

We calculated the rate of change in average choroidal thickness before and after treatment in the 9 mm subfield and the 9–18 mm subfield and compared whether there was a difference between the subfields.

As described above, we determined whether a dominant side exists for the deep choroidal vein and the vortex veins. If either the upper or lower direction was dominant, it was defined as a dominant pattern; otherwise, it was defined as a non-dominant pattern. The choroidal thickness decreased in both the supratemporal and infratemporal square subfields in all three groups, and there was no significant difference in the ratio between them (Table 6).

**Figure 5:**
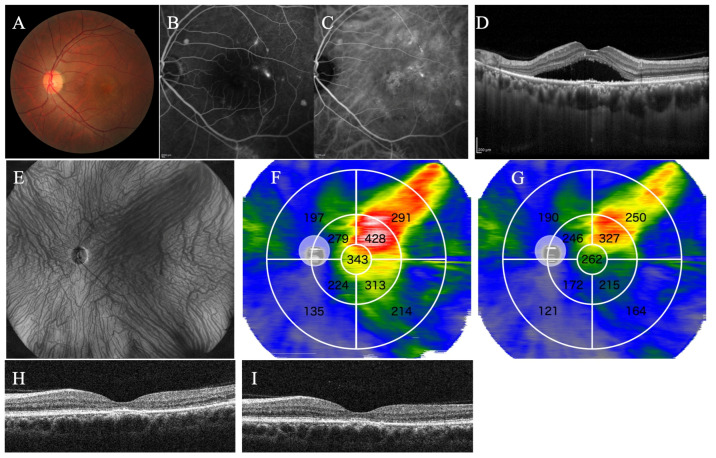
Representative case 1. A 44-year-old male with PNV treated with half-fluence photodynamic therapy combined with intravitreal aflibercept injection. The fundus photograph (**A**), the fluorescein angiography (**B**), and the indocyanine-green angiography before treatment (**C**). (**D**) Type 1 MNV and SRF exist in the macula. (**E**) The en-face image before treatment showed overall dilated choroidal blood vessels. (**F**) In the choroidal thickness map before treatment, the 3–9 and 9–18 mm subfields were divided into four subfields. In the choroidal thickness map, the choroid is displayed in the order of thinness as blue, green, yellow, red, and white. The value of each subfield indicates the mean choroidal thickness (μm) for the subfield and the average choroidal thickness in each subfield was automatically measured. (**G**) The choroidal thickness map 3 months after treatment shows an overall choroid thinner than in (**F**). The SRF that existed before treatment disappeared 1 month after treatment (**H**) and did not recur 3 months after treatment (**I**).

**Figure 6:**
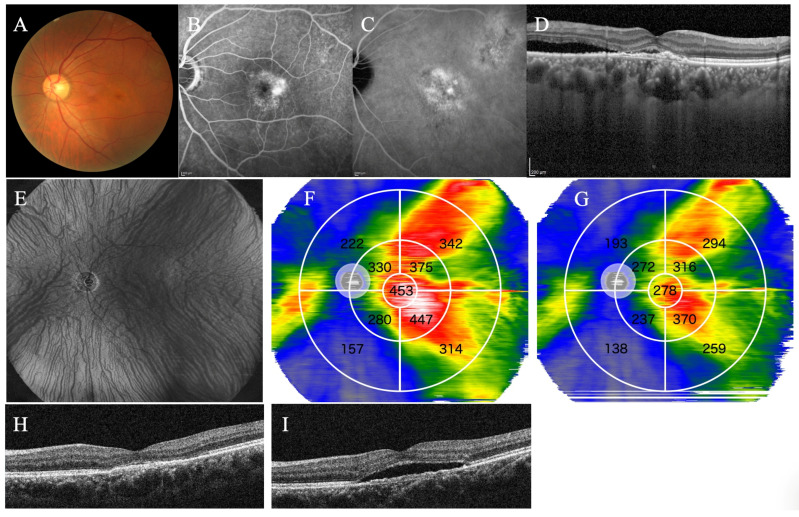
Representative case 2. A 55-year-old male with PNV treated with half-fluence photodynamic therapy combined with intravitreal aflibercept injection. The fundus photograph (**A**), the fluorescein angiography (**B**), and the indocyanine-green angiography before treatment (**C**). (**D**) Type 1 MNV and SRF exist in the macula. (**E**) The en-face image before treatment showed overall dilated choroidal blood vessels. (**F**) In the choroidal thickness map before treatment, the 3–9 and 9–18 mm subfields were divided into four subfields. In the choroidal thickness map, the choroid is displayed in the order of thinness as blue, green, yellow, red, and white. The value of each subfield indicates the mean choroidal thickness (μm) for the subfield, and the average choroidal thickness in each subfield was automatically measured. (**G**) The choroidal thickness map 3 months after treatment shows an overall choroid thinner than in (**F**). The SRF that existed before treatment disappeared 1 month after treatment (**H**) but recurred 3 months after treatment (**I**).

A representative case without recurrence of SRF within 3 months after treatment is shown in Figure 5, while a representative case with recurrence of SRF within 3 months after treatment is shown in Figure 6.

## 4. Discussion

This study demonstrated that half-fluence PDT with adjuvant aflibercept reduced choroidal thickness in both the central and peripheral areas using UWF-OCT analyses. Furthermore, the choroid was thinner in the central area than in the peripheral area. This is the first report to compare choroidal thickness in the central and peripheral regions using UWF-OCT in PNV patients. Moreover, unlike recently shown in CSC patients [23], there was no significant relationship between the reduction in choroidal thickness and the dominant side of the vortex vein.

We previously reported that the choroid was thicker in both the central and peripheral areas in PNV compared to that in typical AMD and that the increased choroidal thickness was pronounced, particularly in the central area [5]. Although the underlying mechanism for the reduction in choroidal thickness following PDT is not fully understood, our results suggest that the thickened choroid in PNV can be improved by PDT.

PDT addresses the underlying pachychoroid-based pathology rather than providing simple photocoagulation at the leakage points, as it has been shown to improve choroidal thickening and CVH in CSC [15]. The dynamic changes in choroidal blood circulation following PDT in PNV may shed light on its pathology. Accordingly, we analyzed the changes in choroidal thickness post-PDT using UWF-OCT. 

In this study, the areas irradiated with PDT were located near the macula. It has been hypothesized that congestion in the vortex vein plays a role in the pathogenesis of CSC and PNV [25,26,27]. Consistently, various studies have reported a vertical asymmetry in the running pattern of deep choroidal veins in PSD [20,27,28]. To investigate the relationship between the dominant side of choroidal outflow from the macula and choroidal thickness, we identified the dominant side based on the location of the watershed zone. However, no correlation was observed between the reduction in choroidal thickness after PDT and the dominant side of the vortex vein. The decrease in choroidal thickness on the dominant side was not significantly different from that on the non-dominant side. This finding contrasts with those of previous studies on patients with CSC treated with PDT [23]. Further research, including a larger cohort of patients with PNV treated with PDT, is warranted. Additionally, evaluating the volumetric changes in the choroidal stroma versus the lumen post-PDT is crucial.

PDT may cause the occlusion of the choriocapillaris [29], potentially causing choroidal thinning due to reduced blood flow from the choriocapillaris in the irradiated area. Although PDT was performed around the macular areas where the choroidal blood flowed to the dominant side, the choroidal thickness on the non-dominant side also decreased. Matsumoto et al. reported thinning of the choroid on the dominant side rather than on the non-dominant side after PDT in CSC [23]; however, our results in PNV cases were not consistent with those in CSC. A decrease in choriocapillaris blood flow occurred within the PDT-irradiated area [30]. Given that PDT is performed around the macular area, the choriocapillary blood in the irradiated area should outflow to the vortex vein on the dominant side. Therefore, one might theoretically believe that deep choroidal blood flow decreases mainly on the dominant side [23]. However, choroidal vessels are intertwined and connected throughout the eyeball; therefore, it is not surprising that a reduction in luminal volume of the choroidal veins may occur throughout the choroid. An alternative hypothesis is that PDT could improve CVH through pathways other than the occlusion of the choriocapillaris, such as modifying growth factors (e.g., VEGF) or cytokine/chemokine signaling within the choroid [31,32] and reducing its stromal and luminal volume.

Additionally, we investigated the factors associated with treatment outcomes after PDT and found no statistically significant factors. However, patients with remaining SRF tended to be older than those without SRF (*p* = 0.09). Therefore, older patients with PNV might exhibit greater resistance to PDT in ameliorating SRF. In addition, although there was no significant difference, the choroid in the 9–18 mm subfield before treatment was thinner in the group with remaining SRF at 3 months after treatment (Table 5). This means that in the group with the remaining SRF, the center tended to be relatively thicker than the periphery before treatment. In this study, seven eyes (43.8%) had SRF 3 months after PDT. The proportion of eyes with persistent SRF was higher than that previously reported in patients with CSC [33,34]. Given that some cases of PNV can occur secondary to CSC [3,35], younger patients with PNV may share similarities with patients with CSC in terms of fluid resolution by PDT. In contrast, PNV with a longer clinical course may be associated with RPE dysfunction, which is an important pathology of AMD. Future studies with a larger number of patients treated with PDT are required to clarify this further. Indeed, the mean age in the persistent SRF group tended to be older than that in the non-persistent SRF group (Table 2). Therefore, cases with remaining SRF after treatment might be associated with a relatively long course of PNV, potentially implying the existence of quiescent PNV. For such patients, the effectiveness of PDT might be limited compared to younger PNV patients. 

Seven eyes (43.8%) exhibited a recurrence of SRF. If full-fluence PDT had been performed instead of half-fluence PDT, the recurrence of SRF might have been reduced. However, considering that there were no cases where BCVA increased by 0.2 or more, we assume that patients might have avoided complications by receiving treatment with half-fluence PDT.

In this study, a single dose of aflibercept was administered prior to half-fluence PDT, which requires consideration of the residual effects of aflibercept on decreasing choroidal thickness. A single dose of aflibercept can reduce choroidal thickness, with a peak effect observed at 15 days post-injection [36]. However, there is no clear evidence regarding the extent to which the decreased choroidal thickness returns to the pre-treatment level 3 months after a single dose in AMD. Previous studies involving 144 patients treated with aflibercept showed that choroidal thickness continuously decreased with three loading doses, reducing to an average of 87% of the pretreatment level. The most significant decrease in choroidal thickness occurred 1 month after the first dose, and approximately 40% of the reduction caused by the first injection recovered 2 months after the third dose [37]. Furthermore, during bimonthly injections following the loading phase, fluctuations in choroidal thickness were observed between treatments [37]. These fluctuations indicated that the choroidal thickness nearly returned to its baseline level within 2 months. Therefore, theoretically, the reduction in choroidal thickness is presumed to recover 3 months after a single dose.

This study has some limitations. First, this was a single-center retrospective study with a relatively small number of patients receiving half-fluence PDT. A larger sample size may elucidate a clearer relationship between the presence of SRF at 3 months post-treatment and patient age. Findings regarding the patterns of deep choroidal veins and the reduction in choroidal thickness might change with a larger study population. Furthermore, there is no definitive consensus on the diagnostic criteria for PNV yet, and we did not investigate genetic factors in this study. During the observation period of this study, half-fluence PDT combined with intravitreal aflibercept injections were performed for all patients with PNV. However, patients with a history of allergy to contrast agents, rendering them unable to undergo fluorescein/indocyanine-green angiography, were excluded from consideration for PDT treatment. In addition, the follow-up period after PDT was relatively short, so we were unable to discuss the relationship between choroidal thickness reduction and the frequency of retreatments due to fluid recurrence. Further investigation is needed into the clinical utility, such as which features characterize PNV with a favorable treatment response. Although UWF-OCT was performed to analyze choroidal thickness, there were limitations in assessing areas, including the vortex vein ampulla, owing to the limited angle of view. A study analyzing the vortex vein area in patients with PNV would contribute to a better understanding of its pathology in future research.

In conclusion, treatment with half-fluence PDT and aflibercept decreased the entire choroidal thickness in patients with PNV, similar to the finding in CSC cases, and no relationship was observed between reduction in choroidal thickness and the dominant side of the vortex vein, unlike CSC. Our analysis of choroidal thickness using UWF-OCT, focusing on the treatment of PNV, may contribute to unraveling the mechanisms and pathogenesis of PDT in PSD.

## Figures and Tables

**Table 1:** Patients’ characteristics before treatment.

	16 Eyes of 16 Patients, *n* (%) or Mean ± SD
Male	9 (56.3%)
Age (y)	64.9 ± 12.9
Axial length (mm)	24.4 ± 1.1
BCVA (logMAR)	0.15 ± 0.20
GLD (μm)	3506 ± 934
CMT (μm)	336.0 ± 76.0
Hypertension	8 (50.0%)
Diabetes	2 (12.5%)
Dominant sides of deep choroidal veins	
Upper dominant	8 (50.0%)
Lower dominant	3 (18.8%)
Asymmetry	5 (31.3%)

SD: standard deviation; BCVA: best-corrected visual acuity; logMAR: logarithm of the minimum angle of resolution; GLD: greatest linear dimension; CMT: central macular thickness.

**Table 2:** Factors related to the presence of SRF after treatment.

	SRF (−)	SRF (+)	*p*-Value
n	9	7	
Male	6 (66.6%)	4 (56.3%)	0.61
Age (y)	60.1 ± 11.5	71.0 ± 12.5	0.09
Axial length (mm)	24.6 ± 0.7	24.2 ± 1.4	0.46
BCVA (logMAR)	0.16 ± 0.24	0.14 ± 0.18	0.82
GLD (μm)	3644 ± 1036	3329 ± 828	0.52
CMT (μm)	327.3 ± 68.4	347.1 ± 89.2	0.62
Asymmetry of vortex vein			0.86
Upper dominant	4 (44.4%)	4 (57.1%)	
Lower dominant	2 (22.2%)	1 (14.3%)	
Asymmetry	3 (33.3%)	2 (28.6%)	

*p*-values were calculated using Fisher’s exact test for ordinal data and the Mann–Whitney U test for continuous data. SRF: subretinal fluid; BCVA: best-corrected visual acuity; logMAR: logarithm of the minimum angle of resolution; GLD: greatest linear dimension; CMT: central macular thickness.

**Table 3:** Choroidal thickness before and after half-fluence PDT.

Areas	Before Treatment	After Treatment	*p*-Value
3 mm subfield	356.4 ± 63.0	299.4 ± 62.5	<0.001
3–9 mm subfield	313.7 ± 55.2	276.8 ± 56.7	<0.001
Supratemporal 3–9 mm	351.1 ± 61.1	307.2 ± 60.0	<0.001
Infratemporal 3–9 mm	317.8 ± 68.1	274.9 ± 64.4	<0.001
Supranasal 3–9 mm	314.4 ± 75.0	285.4 ± 72.4	<0.001
Infranasal 3–9 mm	271.2 ± 58.0	239.4 ± 63.4	<0.001
9–18 mm subfield	237.5 ± 41.4	216.9 ± 39.9	<0.001
Supratemporal 9–18 mm	285.3 ± 53.9	259.9 ± 50.8	<0.001
Infratemporal 9–18 mm	235.7 ± 52.3	213.4 ± 47.9	<0.001
Supranasal 9–18 mm	253.4 ± 64.2	233.8 ± 61.6	<0.001
Infranasal 9–18 mm	175.6 ± 38.2	160.8 ± 35.2	<0.001

The data are shown as means with standard deviations. *p*-values were calculated using the Wilcoxon signed-rank test to compare choroidal thickness before and after treatment. PDT: photodynamic therapy.

**Table 4:** Ratio of change in choroidal thickness before and after treatment in the posterior and peripheral areas.

Areas	Before Treatment	After Treatment	Ratio	*p*-Value
9 mm subfield	286.5 ± 53.1	251.3 ± 51.0	0.88 ± 0.07	0.002
9–18 mm subfield	237.5 ± 47.1	216.9 ± 39.9	0.91 ± 0.06	

The data are shown as means with standard deviations. *p*-values were calculated using the Wilcoxon signed-rank test.

**Table 5:** Relationship between choroidal thickness before PDT and the presence of SRF after PDT in each subfield.

Areas	SRF (−)	SRF (+)	*p*-Value
3 mm subfield	355.1 ± 50.3	358.0 ± 80.9	0.93
9 mm subfield	296.2 ± 40.1	274.1 ± 60.3	0.39
9–18 mm subfield	252.6 ± 39.9	218.1 ± 37.3	0.09

The data are shown as means with standard deviations. *p*-values were calculated using the Wilcoxon signed-rank test to compare choroidal thickness before and after treatment. PDT: photodynamic therapy.

**Table 6:** Differences in the ratio of change in choroidal thickness due to treatment between the dominant and non-dominant sides.

Asymmetrical Pattern (*n* = 11; Upper Dominant, *n* = 8, Lower Dominant, *n* = 3)				
**Areas**	**Before Treatment**	**After Treatment**	**Ratio**	***p*-Value**
Dominant side	273.5 ± 49.4	257.5 ± 53.0	0.94 ± 0.05	0.68
Non-dominant side	253.2 ± 36.6	237.8 ± 47.7	0.94 ± 0.05	
**Symmetrical pattern (*n* = 5)**				
**Areas**	**Before treatment**	**After treatment**	**Ratio**	***p*-value**
Upper area	253.4 ± 56.0	227.4 ± 60.4	0.89 ± 0.09	0.17
Lower area	186.2 ± 30.8	154.0 ± 40.2	0.84 ± 0.20	

The data are shown as means with standard deviations. *P*-values were calculated using the one-way analysis of variance.

## Data Availability

All data generated or analyzed during this study are included in this article. Further inquiries can be directed to the corresponding author.

## Supplementary Materials

The following supporting information can be downloaded at: https://www.mdpi.com/article/10.3390/jcm13061608/s1, Figure S1: Comparison of the ratio of change in choroidal thickness between subfields.
