# Supplementary material for: Wide-Field Choroidal Thickness Analysis after Half-Fluence Photodynamic Therapy Combined with Intravitreal Aflibercept Injection in Pachychoroid Neovasculopathy
Source: J Clin Med. 2024 Mar 11;13(6):1608. doi: 10.3390/jcm13061608 (PMC10971741; doi:10.3390/jcm13061608)
Supplement: Supplementary file 1 [file jcm-13-01608-s001.zip › jcm-2868884-supplementary.pdf]

Journal of Clinical Medicine

MDPI

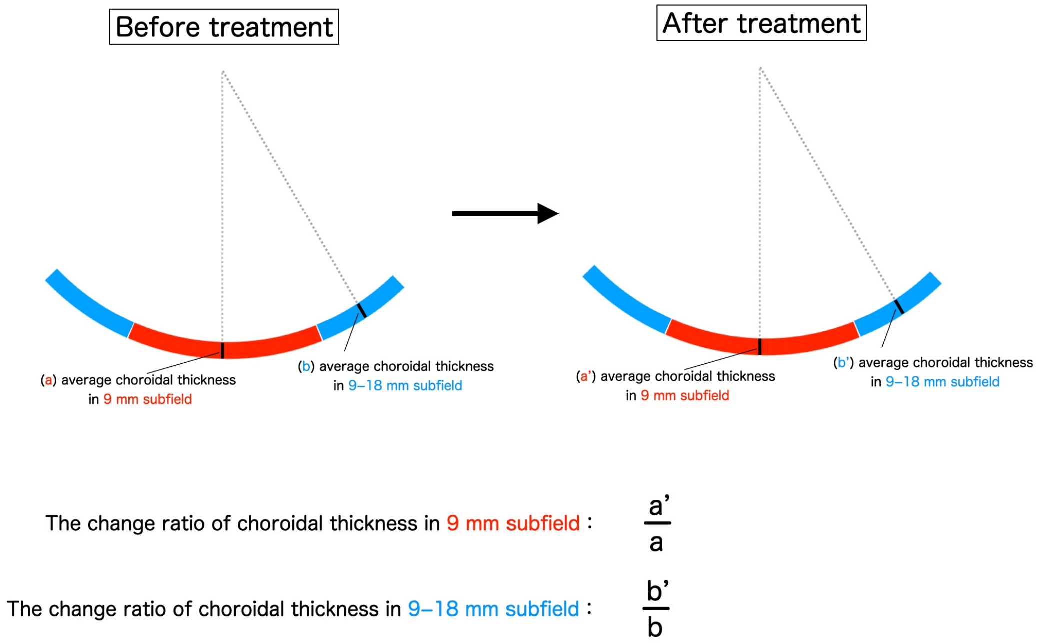

$$\frac{a'}{a}$$

$$\frac{b'}{b}$$

Figure S1. Comparison of the ratio of change in choroidal thickness between subfields.
